# Physical Activity Patterns of Acute Stroke Patients Managed in a Rehabilitation Focused Stroke Unit

**DOI:** 10.1155/2013/438679

**Published:** 2013-08-19

**Authors:** Tanya West, Julie Bernhardt

**Affiliations:** ^1^School of Health Sciences, La Trobe University, Melbourne, VIC 3086, Australia; ^2^Physiotherapy Department, Royal Perth Hospital, Perth, WA 6000, Australia; ^3^Stroke Division, Florey Institute of Neuroscience and Mental Health, Melbourne, VIC 3084, Australia

## Abstract

*Background*. Comprehensive stroke unit care, incorporating acute care and rehabilitation, may promote early physical activity after stroke. However, previous information regarding physical activity specific to the acute phase of stroke and the comprehensive stroke unit setting is limited to one stroke unit. This study describes the physical activity undertaken by patients within 14 days after stroke admitted to a comprehensive stroke unit. *Methods*. This study was a prospective observational study. Behavioural mapping was used to determine the proportion of the day spent in different activities. Therapist reports were used to determine the amount of formal therapy received on the day of observation. The timing of commencement of activity out of bed was obtained from the medical records. *Results*. On average, patients spent 45% (SD 25) of the day in some form of physical activity and received 58 (SD 34) minutes per day of physiotherapy and occupational therapy combined. Mean time to first mobilisation out of bed was 46 (SD 32) hours post-stroke. *Conclusions*. This study suggests that commencement of physical activity occurs earlier and physical activity is at a higher level early after stroke in this comprehensive stroke unit, when compared to studies of other acute stroke models of care.

## 1. Introduction

Current stroke guidelines recommend increased physical activity early after stroke [1–3]. Furthermore, favourable outcomes have been reported for stroke unit care in which patients commence frequent out of bed activity within 24 hours of stroke [4], and preliminary evidence to support this intervention has emerged from two small randomised controlled trials [5, 6]. Nevertheless, in a previous systematic review we found that, in spite of inconsistencies in the classification of physical activity across studies, hospitalised stroke patients consistently spend large proportions of the day inactive and this lack of physical activity appears pronounced within the first 14 days after stroke [7]. Few researchers have investigated physical activity in the acute phase of stroke. Only three studies in our review specifically investigated patients within 14 days after stroke [8–10] and a median of 65% of the day was spent physically inactive across the patient groups in these three studies [7]. However, in studies of patients within 14 days after stroke managed in a stroke unit that incorporates rehabilitation (a so-called comprehensive stroke unit (CSU)) in Trondheim, Norway, patients were found to spend an average of only 30–40% of the day physically inactive [8, 11]. 

Unlike acute stroke units (ASUs) which focus primarily on acute care, the CSU combines acute care with a rehabilitation focus. Patients are admitted acutely but also receive rehabilitation that may last for several weeks if required [12]. Early physical activity has been described as a key component of the CSU model of care [4, 13]. In a review of the literature describing the ASU and CSU models of care we found an emphasis on acute medical management, increased nurse staffing, early assessment and investigation, and intensive physiological monitoring in ASU care models [14]. In contrast, CSU care tended to have a greater emphasis on multidisciplinary teamwork and involvement in rehabilitation, active participation of the patient and family, and early mobilisation policies [14]. These underlying features of the CSU may facilitate increased physical activity early after stroke compared to alternative models of acute stroke care. However, with the exception of the Trondheim CSU, little information exists regarding early physical activity in CSU care. There is a need to determine if the level of early physical activity reported in the Trondheim CSU is replicated in other CSUs.

The purpose of this study was to describe the physical activity undertaken by patients within 14 days after stroke admitted to a CSU. The primary aim of the study was to determine how much physical activity is undertaken during the working day by patients within 14 days after stroke admitted to a CSU. Secondary aims were to determine where and with whom this activity took place; the amount of formal therapy received and the level of patient activity during formal therapy; when patients first commenced physical activity out of bed and who initiated this. 

## 2. Methods

### 2.1. Study Design and Participants

This study was a prospective observational study conducted in the 14-bed CSU of the Royal Perth Hospital, a large metropolitan tertiary hospital in Perth, Western Australia. In accordance with the Stroke Unit Trialists' Collaboration definitions of the CSU models of care [12], the Perth CSU admitted patients acutely and combined both acute care and rehabilitation. Rehabilitation was provided simultaneously as part of the acute management and ongoing rehabilitation could be provided for as long as necessary on the stroke unit; however most patients requiring inpatient rehabilitation beyond a few weeks were usually transferred to a rehabilitation facility at another site. 

Patients included in the study were aged 18 years or over, with a diagnosis of stroke, who were admitted to the stroke unit and were within 14 days of stroke onset. Patients were recruited from the beginning of 2008 to the end of 2010. Patients were excluded from the study if they were receiving palliative care or if discharge was planned prior to completion of the day of behavioural observation. Ethical approval for this study was obtained from the Faculty of Health Sciences Human Ethics Committee at La Trobe University and the Royal Perth Hospital Ethics Committee. Informed consent was obtained from all participants or a responsible third party where the patient was unable to provide consent themselves.

### 2.2. Behavioural Mapping

Physical activity, location, and people present were recorded across the day for each patient using established standardised behavioural mapping procedures, which have been previously demonstrated to have high interrater reliability [9]. Behavioural mapping was carried out over the working day, defined as a nine-hour period between 8 am and 5 pm on a weekday, when the patients were considered to be most active. Up to 10 patients could be mapped on each day of observation. Observations took place at 10-minute intervals with the exception of up to five randomly scheduled 10-minute rest periods for the observer. High consistency of patient behaviour across weekdays has been reported in a previous study [8]; therefore, patients were observed for a single working day; however the weekday chosen for each day of observation varied across the duration of the study. To accommodate the desire to obtain a large sample of patients with a wide range of stroke related disability, it was planned that observation would be undertaken approximately every six to eight weeks, depending on the availability of suitable patients. Up to 10 patients could be mapped on each day of observation. Patients and staff were informed that patient activity was being monitored; however they were instructed that they should not alter their usual behaviour. Wherever possible the observer attempted to remain inconspicuous to avoid influencing behaviour.

Physical activity was grouped into the following five categories based on previous activity definitions [9]:

- nil physical activity: lying in bed inactive,
- nonphysical activity: passive activities while resting in bed including reading, watching TV, talking, and eating,
- low physical activity: sitting supported out of bed, hoist transfers,
- moderate physical activity: sitting unsupported, transfers with feet on floor,
- high physical activity: standing, walking, and stair climbing.

### 2.3. Therapist Report

The amount and type of patient activity undertaken during formal physiotherapy and occupational therapy sessions on the day of observation were recorded in minutes by the treating therapists on a recording form. This provided more detailed information about patient activity during formal therapy sessions. The validity of this method of therapist report has been established previously [15]. 

### 2.4. Commencement of Physical Activity

The time to the patients' first mobilisation out of bed both from the time of stroke onset and from the time of hospital admission was derived from the patients' medical records. The people involved in this first mobilisation were also identified from the medical records.

### 2.5. Patient Characteristics

Demographic data and information regarding the patient's stroke were obtained from the patient's medical notes. Premorbid function was measured using the modified Rankin Scale (mRS) [16]. Type of stroke was classified according to the Oxfordshire Community Stroke Program (OCSP) classification [17]. Stroke severity was determined using the National Institutes of Health Stroke Scale (NIHSS) [18] from a retrospective review of the medical records [19]. The patient's motor function on the day of observation was assessed by the treating physiotherapist using the Mobility Scale for Acute Stroke (MSAS) [20]. The gait score from this scale was used to group patients into independent (MSAS gait = 6) or dependent (MSAS gait < 6) ambulation categories.

### 2.6. Data Analysis

The average proportion of the working day which patients spent in each activity category, each location and with different people present was calculated. In order to determine where and with whom patients were most active, the proportion of observations recorded in each activity category was calculated for different locations, and for different people. To examine multidisciplinary and family involvement in therapy, the proportion of observations recorded with both therapy staff and other people present was calculated. To determine where therapy took place the proportion of observations recorded with therapy staff in different locations was calculated.

The therapist report data was used to determine the proportion of patients treated by physiotherapists and occupational therapists. Means were calculated for the number of therapy sessions per day, minutes of therapy per day, minutes per therapy session, and the proportion of therapy time spent in each activity category. 

The mean time to first mobilisation out of bed and the proportion of patients mobilised within 12, 24, and 48 hours were calculated, as well as the proportion of patients first mobilised by different health professionals.

## 3. Results

### 3.1. Patient Characteristics

A total of 139 patients were recruited to the study. Nine of these patients were part of a randomised controlled trial examining early mobilisation and were therefore excluded from the data analysis. The characteristics of the remaining 130 patients are presented in Table 1.

### 3.2. Physical Activity

**Figure 1:**
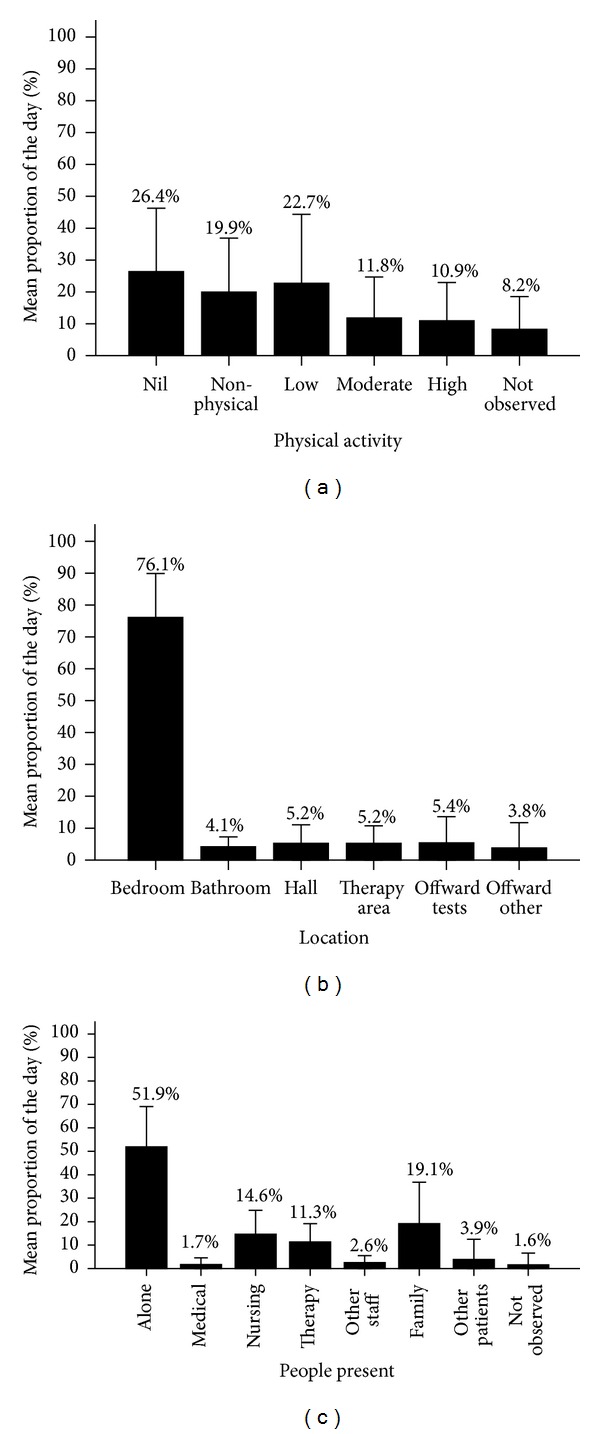
Mean (SD) proportion of the day (a) in each physical activity category, (b) in each location, and (c) with different people present. Therapy includes physiotherapy, occupational therapy, and speech therapy. People present categories are not mutually exclusive.

The mean proportion of time spent in each physical activity level, in each location, and with different people present is illustrated in Figure 1. On average, patients spent 45% (SD 25) of the day involved in some form of physical activity out of bed, including 22% (SD 21) of the day in moderate or high physical activity. Patients spent an average of 46% (SD 24) of the day in the nil or nonphysical activity category. 

### 3.3. People Present and Location of Activity

Most of the day was spent in the bedroom area (Figure 1(b)), and in 60% of the observations recorded in the bedroom patients were inactive or involved in passive activities, such as talking, reading or watching TV in bed. In contrast, when patients were in therapy areas, bathrooms, hallways, and offward for purposes other than investigations, they were inactive or involved in nonphysical activity in less than 10% of these observations. 

On average, patients were alone for more than 50% of the day, and when not alone nursing staff and family were the people most often present (Figure 1(c)). However, patients were least active when observed alone, with nursing staff or with family, spending more than half of these observations inactive or not involved in physical activity. 

In less than 1% of observations patients were observed with both a therapist and someone else present (physiotherapist/nurse 0.3%; physiotherapist/family 0.5%; occupational therapist/nurse 0.1%; occupational therapist/family 0.7%). Physiotherapy and occupational therapy staffs were both present at the same time for only 0.1% of observations. Patients also spent very little time with therapists in ward areas including the bedroom, bathroom, and hallway. Only 2.8% of observations were recorded with a physiotherapist while in a ward area and 2.6% with an occupational therapist while in a ward area.

### 3.4. Therapy Activity

Most patients received at least one session of physiotherapy and one session of occupational therapy per day, averaging approximately one hour per day of therapy from these two disciplines combined (Table 2). 

**Figure 2:**
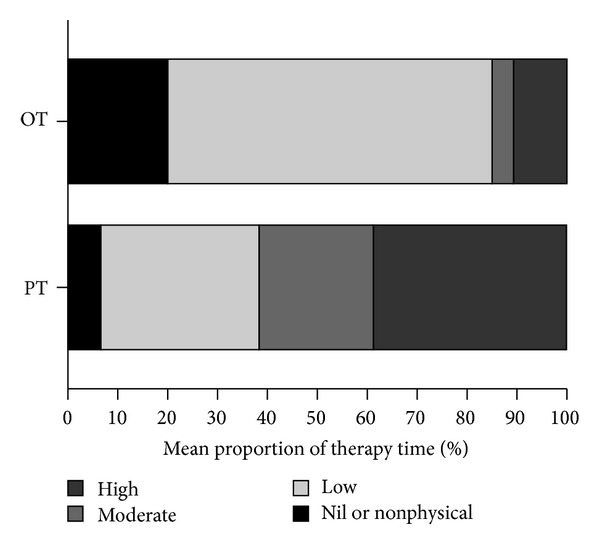
Mean proportion of formal therapy time in each physical activity category for physiotherapy (PT) and occupational therapy (OT).

Figure 2 illustrates the level of physical activity undertaken during physiotherapy and occupational therapy sessions. Patients were engaged in moderate to high level activities for an average of 61% (SD 31) of physiotherapy time and in low level physical activity for an average of 65% (SD 42) of occupational therapy time. 

### 3.5. First Mobilisation out of Bed

**Figure 3:**
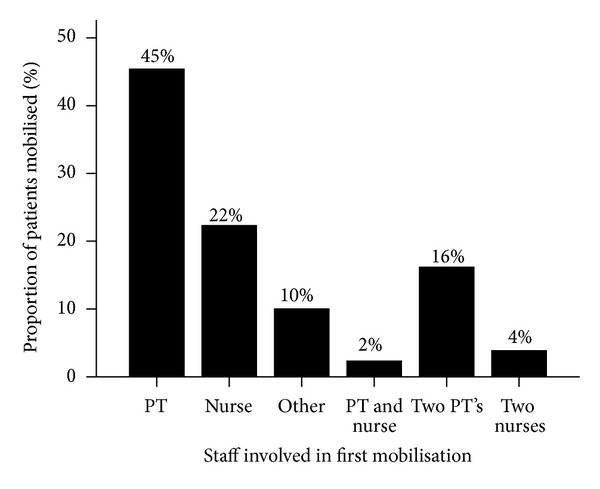
Proportion of patients first mobilised by different staff.

The time to first mobilisation is presented in Table 3, while Figure 3 depicts which people assisted with the first mobilisation out of bed. The average time to first mobilisation out of bed was 31 hours from admission and 46 hours after stroke. In total, physiotherapy staff were involved in 63% of first mobilisations and nursing staff assisted in 28%. 

## 4. Discussion

This study examines patient activity after stroke in a CSU, including physical activity across the day, the location of activity and the people involved, therapy specific activity, and the timing of commencement of physical activity. Few other previous studies have examined physical activity specifically in the first 14 days after stroke [8–11], and in all but one of these studies [11] sample size was small and the timing of commencement of physical activity was not reported. Furthermore, early physical activity has previously only been examined in one other CSU, based in Trondheim, Norway [8, 11]. Given the emerging literature in support of early physical activity after stroke and the potential for the CSU model to promote early physical activity, this study expands previous knowledge regarding early physical activity after stroke particularly in relation to the CSU and the timing of commencement of physical activity. In addition, the standardised behavioural mapping procedure and classification of physical activity employed in the current study have previously been used in a number of previous studies of early physical activity [8, 9, 11]. The data collection form used for the behavioural mapping procedures is available from the author. This standardisation of methodology overcomes the issues of inconsistency across physical activity studies which we identified in our previous systematic review [7], thereby allowing for better comparison and pooling of data with other studies. 

The findings of this study indicate that patients in the Royal Perth Hospital CSU are involved in some form of physical activity out of bed for almost half the day, including nearly a quarter of the day in moderate or high level activity. Furthermore, patients usually commence activity out of bed within 48 hours after stroke and participate in an average of approximately one hour per day of occupational therapy and physiotherapy combined. In comparison to previous studies of early activity in acute stroke units (ASUs) in Australia [9] and general medical wards and an ASU in Europe [10], the patient activity levels in this Perth CSU are higher. 

Increased early activity in the Trondheim CSU has previously been attributed to an increased focus on early intensive rehabilitation, policies, and procedures which promote early mobilisation and avoidance of bed rest, training and extensive involvement of nursing staff in early mobilisation, increased staffing, and a physical environment which encourages activity [8]. The Perth CSU shares similar characteristics with the Trondheim CSU which may have contributed to the higher activity levels in the current study when compared to previous studies of early activity in other models of acute stroke care. Staff in the Perth CSU aimed to commence rehabilitation, including mobilisation out of bed, within 24 to 48 hours of admission, and to continue ongoing intensive rehabilitation thereafter. Procedures to manage blood pressure and fluid balance supported the practice of early mobilisation. Staff received education and training in early mobilisation. Bathroom areas were located separately from the bedroom areas, providing opportunities for mobilisation when patients need to be transported to the bathroom. Patients who were unable to ambulate with or without assistance were each provided with their own wheelchair, allowing them to sit out of bed as much as tolerated each day and to be easily transported outside of bedroom areas by family and staff. Physiotherapy and occupational therapy areas were located nearby to the ward, and patients generally attended therapy in these areas every weekday. In addition, many patients also participated in therapy sessions away from the ward, including outdoors for the practice of outdoor mobility and separate kitchen areas for the practice of higher level activities of daily living. Perhaps the most noticeable difference between the Perth CSU and the Trondheim CSU is the staffing level. A physiotherapist-patient ratio of 1 : 8 was reported for the Trondheim unit; however occupational therapy was not routinely provided [8]. In comparison the Perth unit had a physiotherapist-patient ratio of approximately 1 : 11 and an occupational therapist-patient ratio of 1 : 13. A nurse-patient ratio of 1 : 3 was reported for the Trondheim unit [8], compared to 1 : 4 in the Perth unit. 

Despite the higher level of early physical activity found in the CSU in this study in comparison to other models of acute stroke care, patients in the Perth CSU were still less active than those in the Trondheim CSU, where patients spent almost 70% of the day engaged in physical activity out of bed [11]. This indicates that activity levels could be further increased in the Perth CSU.

In the Perth CSU, patients spent approximately three quarters of the day in their bedroom, where they were less likely to be physically active. Limited time was spent in locations where increased physical activity might be encouraged, such as the hallway, bathroom, and therapy areas. Providing patients with easier access and more opportunities to spend time in areas other than the bedroom may help to increase physical activity. In the Trondheim unit this has been accomplished by providing a communal dining area and passive recreation areas on the ward [11]. In addition to these environmental features, however, the Trondheim unit has previously been described as having a strong emphasis on patient and family involvement in rehabilitation and a team approach to rehabilitation [4], and it is believed that this “culture” is likely to be important in promoting physical activity [11].

In comparison to the Trondheim unit, there appears to be less involvement of nursing staff and family in rehabilitation in the stroke unit in this study, as well as a lack of patient-initiated activity, which may restrict physical activity levels. Patients were alone for a large proportion of the day, and when the patients were not alone they were most often with nursing staff or family. However for more than half the time where patients were alone, with nursing staff or with family, they were inactive or involved in nonphysical activity such as talking, reading, or watching TV in bed. 

Patients in the current study were with nursing staff for 15% of the day. In comparison, patients in the Trondheim unit spent 22% of the day with nursing staff [11]. The reduced time with nursing staff could be at least partially explained by the lower nurse staffing levels in the Perth stroke unit. Less time with nursing staff could contribute to reduced nursing staff involvement in rehabilitation. 

On average, patients in the Perth unit received 35 minutes per day of physiotherapy and 23 minutes per day of occupational therapy. Patients were involved in some form of physical activity for most of this time. Patients in the Trondheim unit received more daily physiotherapy time [11], however occupational therapy is not routinely provided [8]; therefore, differences in the amount of time in therapy cannot explain the differences in the amount of physical activity between the two sites. However, when patients were observed with therapists, there was further evidence of a lack of multidisciplinary input and family involvement in rehabilitation, which may have contributed to lower activity levels in the Perth unit. The therapy areas are located separately from the ward and patients were rarely observed with therapists in ward areas such as the bedroom, bathroom, or hallway. Other staffs or family members were rarely present when physiotherapy or occupational therapy staff were with patients. More time with therapists on the ward and working in conjunction with other staffs or family members may promote greater teamwork between therapy staff and nursing staff and encourage family involvement in rehabilitation. By practicing therapy activities in ward areas patients may also be better able to put into practice these activities outside of therapy time. 

Time to first mobilisation in the Perth unit compares favourably with that reported for stroke patients in other hospitals in Australia [21–23] and overseas [24]. However, more patients were mobilised within 48 hours in one Australian ASU [25] and within 24 hours in the Trondheim CSU [11]. This delay may be at least partially explained by the limited multidisciplinary cooperation in rehabilitation including a reliance on physiotherapy to initiate activity out of bed. Physiotherapists assisted in the first mobilisation out of bed in almost two-thirds of patients in the current study. In comparison, nursing staff were involved in approximately one-third of first mobilisations. Furthermore, physiotherapy and nursing staff worked together to assist in the first mobilisation for only 2% of patients. The reliance on physiotherapy could contribute to a greater time to first mobilisation if physiotherapy staff are not readily available, particularly on weekends and evenings.

A number of limitations need to be acknowledged. Although the observation technique used in this study was standardised and the observers trained prior to commencement, observation has the potential to influence the activity of the staff or patients observed. If this were true, the activity levels in this study are likely to be higher than those seen under usual circumstances. Furthermore, intermittent observation provides only a “snapshot” of patient activity, not continuous measurement of activity. It remains however the only method currently available to capture not just activity, but people assisting and the location of the activity which is very valuable in examining how care is organised. For the purposes of this study, we believe the advantages afforded by observation outweigh the disadvantages. 

A further limitation was the use of the medical record to determine time to first mobilisation. It is possible that staff may have incorrectly documented the time of first mobilisation or may have even failed to document the first mobilisation altogether; therefore the precision of this data may be questionable. As many of the patients were recruited to the study some days after they were first mobilised out of bed, this was the most accurate means we had of acquiring this information. 

Finally, the comparison of the results of our study to previous studies of early physical activity after stroke may be limited by inconsistencies in the classification of physical activity and the possible heterogeneity of patient populations across studies. Although the same model of behavioural mapping has been employed in a number of studies, notably it is the same as that used in the Trondheim unit; to more accurately compare physical activity levels in a CSU with other acute stroke services, further research is required which provides a direct comparison between services.

## 5. Conclusions

In a stroke unit which combines acute care with a rehabilitation focus (CSU), patients within 14 days after stroke spend 45% of the day engaged in some form of physical activity. While this compares favourably with other reports from units which focus on acute care alone, in this study few episodes of cooperation between therapist, nurses, or family were observed. Improvement in cooperation may see even higher levels of patient activity in the future. Whether these higher levels of physical activity in the CSU make important contributions to recovery is currently unknown, and at present there is insufficient evidence to determine if the CSU is the optimal model of acute stroke care. Therefore, further research examining the key features of the CSU model, including early physical activity after stroke is required and should also include an evaluation of patient outcome.

## Figures and Tables

**Table 1:** Patient characteristics.

*N*	130
Age	
Mean (SD)	68.3 (13.8)
Gender—*n* (%)	
Male	91 (70.0)
Female	39 (30.0)
First stroke—*n* (%)	
Yes	98 (75.4)
No	32 (24.6)
Days post-stroke at observation	
Mean (SD)	6.9 (3.4)
Stroke type—*n* (%)	
Infarct	112 (86.2)
Haemorrhage	18 (13.8)
NIHSS—*n* (%)	
Mean (SD)	10.3 (7.6)
OCSP infarct classification—*n* (%)	
TACI	39 (30.0)
PACI	29 (22.3)
POCI	11 (8.5)
LACI	33 (25.4)
Side of lesion—*n* (%)	
Left	57 (43.8)
Right	65 (50.0)
Brainstem	5 (3.8)
None evident/unknown	3 (2.3)
Premorbid MRS—*n* (%)	
Independent (0–2)	114 (87.7)
Dependent (>2)	16 (12.3)
Prestroke accommodation—*n* (%)	
Home alone	42 (32.3)
Home with someone	83 (63.8)
Residential care	3 (2.3)
Other	2 (1.5)
Pre-stroke mobility—*n* (%)	
Independent no aids	120 (92.3)
Independent with aid	10 (7.7)
MSAS gait score at observation—*n* (%)	
Independent (=6)	44 (33.8)
Not independent (<6)	86 (66.2)

NIHSS: National Institutes of Health Stroke Scale; OCSP: Oxfordshire Community Stroke Project; TACI: total anterior circulation infarct; PACI: partial anterior circulation infarct; POCI: posterior circulation infarct; LACI: lacunar infarct; MRS: modified Rankin Score; MSAS: mobility scale for acute stroke patients.

**Table 2:** Amount of therapy provided.

	PT	OT	PT and/or OT
Patients treated—*n* (%)	107 (82.3)	85 (65.4)	119 (91.5)
Total number of reported therapy sessions	118	94	212
Number of therapy sessions per day—mean (SD)	0.9 (0.5)	0.7 (0.6)	1.6 (0.8)
Therapy time (mins) per day—mean (SD)	34.8 (24.0)	23.1 (25.6)	57.8 (33.7)
Therapy time (mins) per session—mean (SD)	38.3 (18.1)	31.9 (19.3)	35.5 (18.9)
Frequency of therapy sessions per day—*n* (%)			
None	23 (17.7)	45 (34.6)	11 (8.5)
One	96 (73.8)	76 (58.5)	38 (29.2)
Two	11 (8.5)	9 (6.9)	70 (53.8)
Three	0 (0.0)	0 (0.0)	10 (7.7)
Four	0 (0.0)	0 (0.0)	1 (0.8)

PT: physiotherapy; OT: occupational therapy.

**Table 3:** Time to first mobilisation.

Stroke to admission (hrs)	
Mean (SD)	15.0 (20.5)
Stroke to mobilization (hrs)	
Mean (SD)	46.3 (32.4)
Admission to mobilisation (hrs)	
Mean (SD)	31.3 (27.7)
Stroke to mobilisation—*n* (%)*	
<12 hrs—*n* (%)	8 (6.2)
<24 hrs—*n* (%)	28 (21.5)
<48 hrs—*n* (%)	80 (61.5)
>48 hrs—*n* (%)	50 (38.5)
Admission to mobilisation—*n* (%)*	
<12 hrs—*n* (%)	25 (19.2)
<24 hrs—*n* (%)	67 (51.5)
<48 hrs—*n* (%)	104 (80.0)
>48 hrs—*n* (%)	26 (20.0)

*Cumulative totals.
